# Supplementary material for: A frontal transcallosal inhibition loop mediates interhemispheric balance in visuospatial processing
Source: Nat Commun. 2023 Aug 25;14:5213. doi: 10.1038/s41467-023-40985-5 (PMC10457336; doi:10.1038/s41467-023-40985-5)
Supplement: Supplementary file 1 — Supplementary information [file 41467_2023_40985_MOESM1_ESM.pdf]

Supplementary Information

A Frontal Transcallosal Inhibition Loop Mediates Interhemispheric Balance in Visuospatial Processing

Yanjie Wang†, Zhaonan Chen†, Guofen Ma†, Lizhao Wang, Yanmei Liu, Meiling Qin, Xiang Fei, Yifan Wu, Min Xu*, Siyu Zhang*

*Corresponding author. E-mail: mxu@ion.ac.cn; zhang_siyu@sjtu.edu.cn

Supplementary Figures and legends

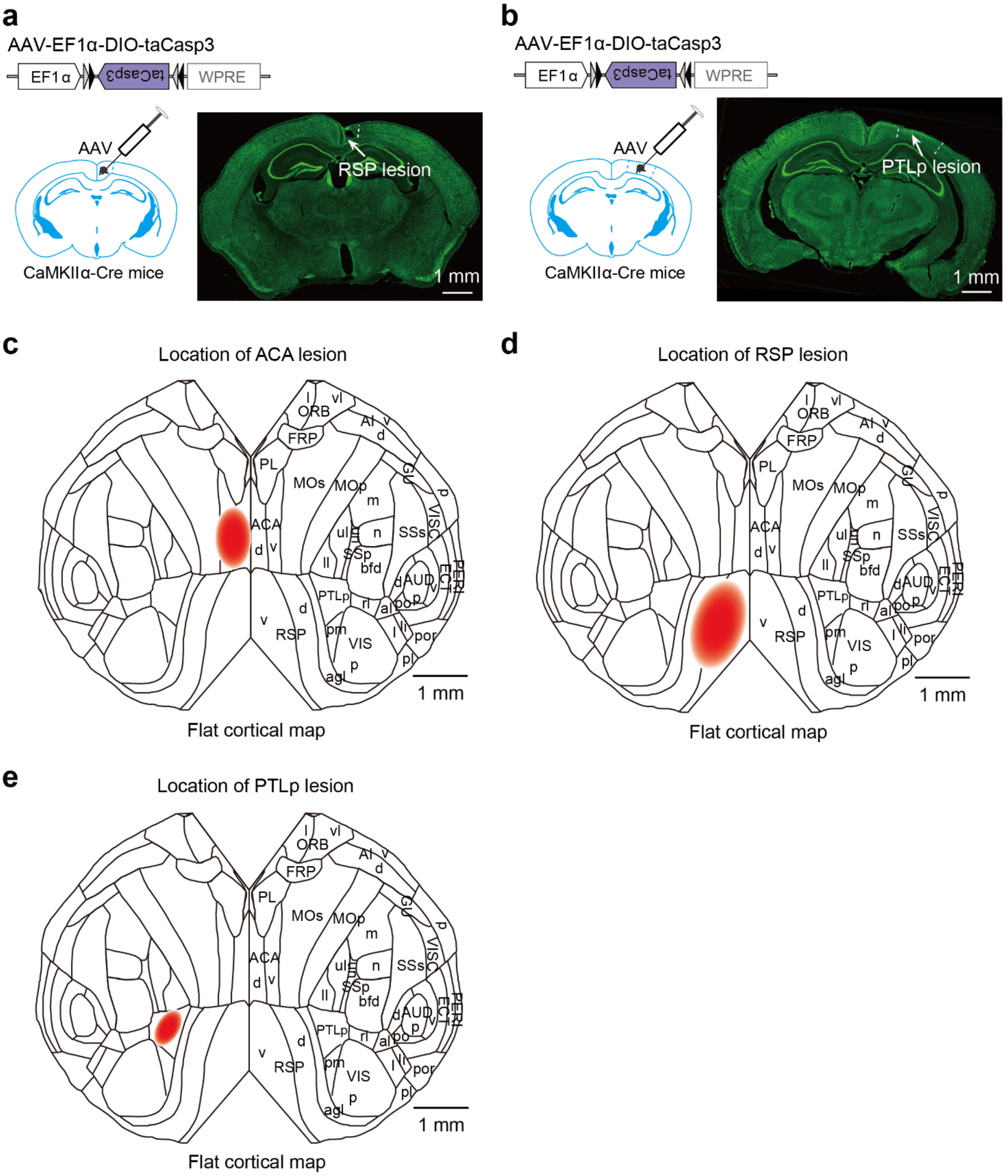

Supplementary Fig. 1. Unilateral lesions of ACA, RSP, and PTLp.

(a) Schematic diagram for the viral strategy to induce unilateral RSP lesion (left) and fluorescence image of a coronal section showing a RSP lesion (right). Green, fluorescent Nissl staining. **(b)** Similar to (a), but for PTLp lesion. **(c)** Location of ACA lesion on a flat cortical map. **(d)** Similar to (c), but for RSP lesion. **(e)** Similar to (c), but for PTLp lesion.

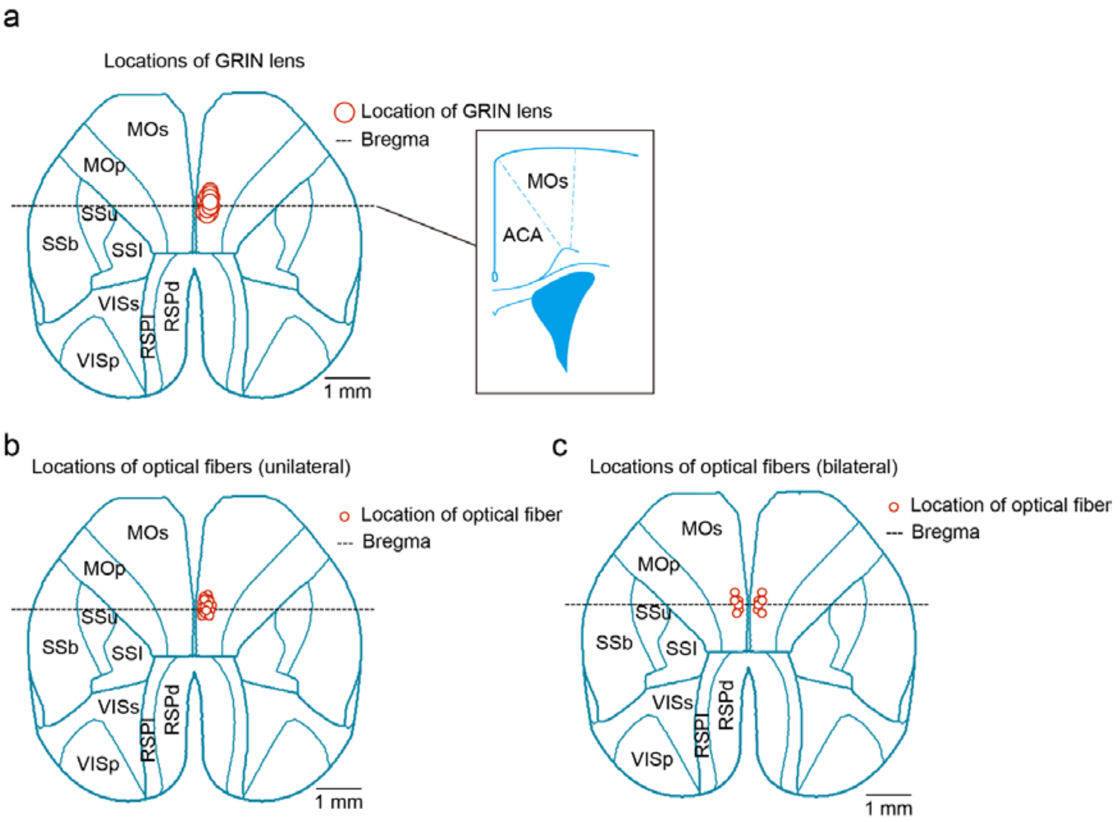

Supplementary Fig. 2. Locations of GRIN lens and optical fibers in the ACA.
(a) Top-down view of a brain atlas illustrating the implantation sites of the GRIN lenses used in the calcium imaging experiments. Inset: A coronal section from the atlas, showing the anatomical locations of the ACA and the secondary motor cortex (MOs) at the level of the bregma. **(b)** Similar to (a), but for the implantation sites of the optical fibers used in the unilateral optogenetic manipulation experiments. **(c)** Similar to (a), but for the implantation sites of the optical fibers used in the bilateral optogenetic manipulation experiments.

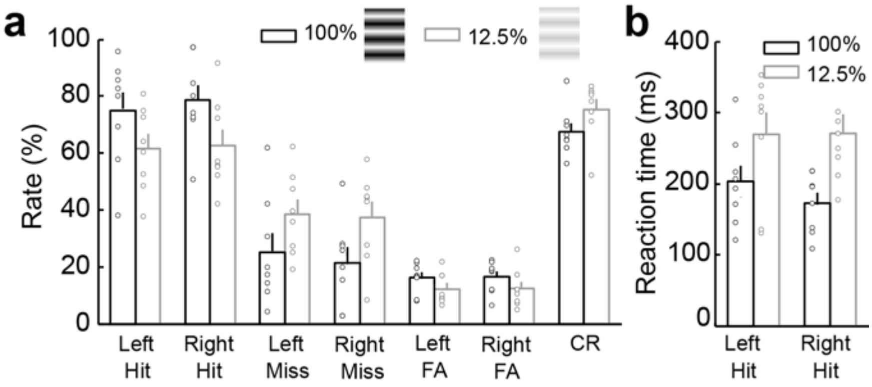

Supplementary Fig. 3. Behavioral performance of mice used for CPN calcium imaging.
(a) Mean left-hit, right-hit, left-miss, right-miss, left-FA, right-FA and CR rates of all mice used for CPN calcium imaging (*n* = 8 mice, 8 session). Black bars, 100% contrast; gray bars, 12.5% contrast. At each contrast level, left-hit + left-miss = 100%; right-hit + right-miss = 100%; left-FA + right-FA + CR = 100%. **(b)** Reaction times in left-hit and right-hit trials at the 100% (black) and 12.5% (gray) contrast levels. Two-way repeated-measures ANOVA of the hit rate, FA rate, and reaction time revealed a significant effect for the contrast level, no significant effect for the left/right spatial location, and no significant interaction between contrast condition and spatial location (hit rate, $F_{side}(1,7) = 0.47$, $P_{side} = 0.52$; $F_{contrast}(1,7) = 5.66$, $P_{contrast} = 0.049$; $F_{side*contrast}(1,7) = 0.84$, $P_{side*contrast} = 0.39$; FA rate, $F_{side}(1,7) = 0.10$, $P_{side} = 0.76$; $F_{contrast}(1,7) = 7.50$, $P_{contrast} = 0.03$; $F_{side*contrast}(1,7) = 0.003$, $P_{side*contrast} = 0.96$; reaction time, $F_{side}(1,7) = 0.67$, $P_{side} = 0.44$; $F_{contrast}(1,7) = 13.35$, $P_{contrast} = 0.008$; $F_{side*contrast}(1,7) = 1.36$, $P_{side*contrast} = 0.28$; two-way repeated ANOVA). All data are presented as the mean ± SEM. Also see Supplementary Data 2 for detailed parameters of ANOVA. Source data are provided as a Source Data file.

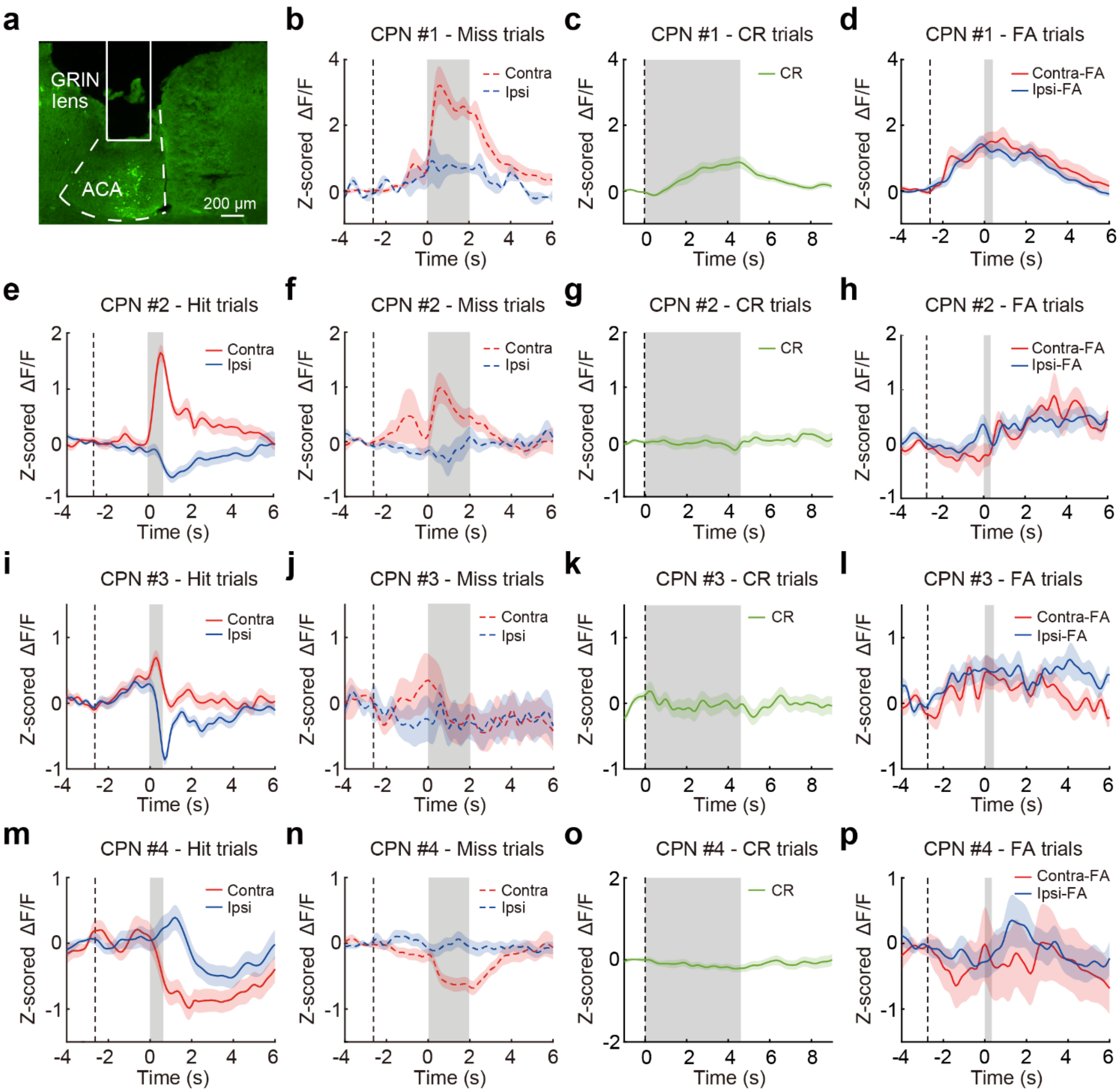

Supplementary Fig. 4. The activity of example CPNs in the 2AUC change-detection task. (**a**) Coronal section showing GCaMP6s fluorescence (green) and the location of implanted GRIN lens. **(b-d)** Averaged ΔF/F (z-scored) of CPN #1. Dashed line, the start of visual white noise. Colored shading, ±SEM. (**b**) Activity in miss trials. Gray shading, window during the presentation of drifting grating. (**c**) Activity in CR trials. Gray shading, window during the presentation of visual white noise. (**d**) Activity in FA trials. Gray shading, window during the wheel rotation. (**e**) Activity in hit trials. Gray shading, window from the start of drifting grating to the end of wheel rotation. **(f-h)** Similar to (**b-d**), but for CPN #2. (**i-l**) Similar to (**e-h**), but for CPN #3. **(m-p)** Similar to (**e-h**), but for CPN #4.

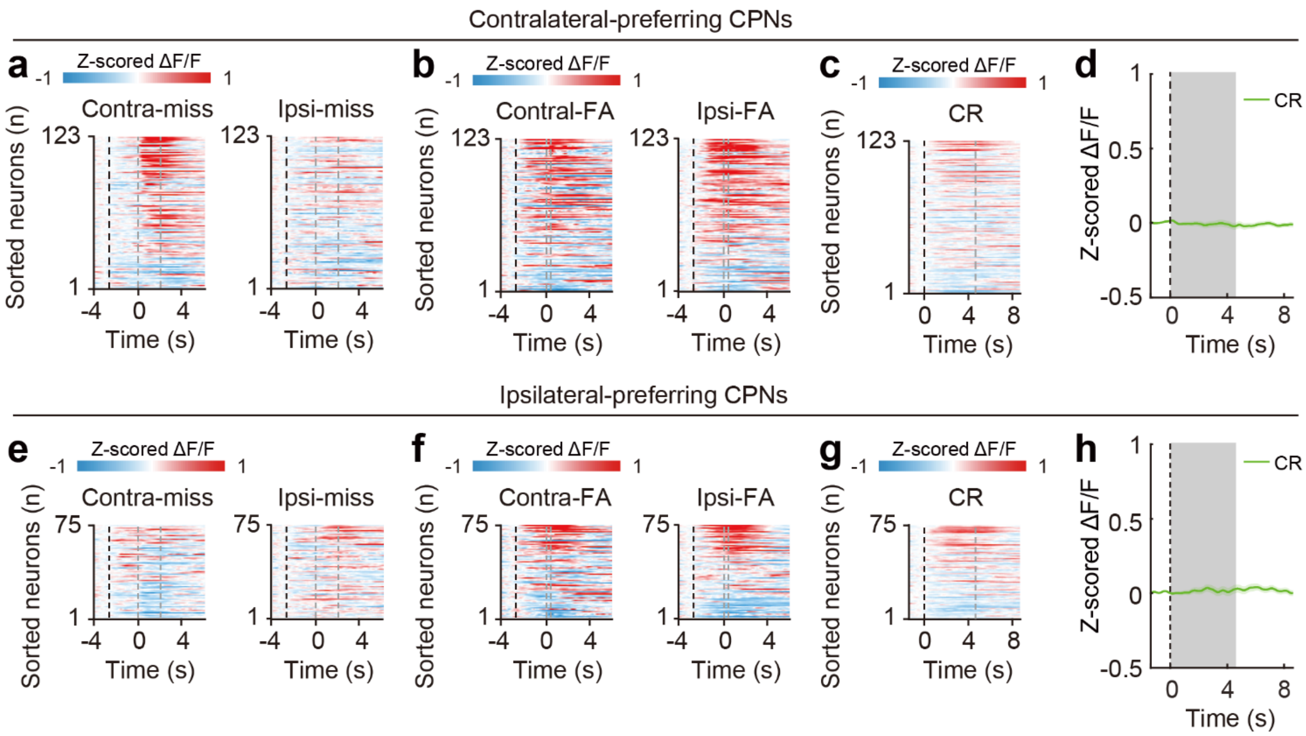

Supplementary Fig. 5. The activity of contralateral-preferring and ipsilateral-preferring CPNs in the 2AUC change-detection task.

(a) Color-coded averaged ΔF/F (z-scored) of contralateral-preferring CPNs in miss trials. Each row shows the average activity of one neuron, sorted by peak response during the visuomotor period in hit trials (same as showing in Fig. 2g). Black dashed line, the start of visual white noise. The left and right gray dashed lines present the start and end of drifting grating, respectively. **(b)** Similar to (a), but for contralateral-preferring CPNs in FA trials. Black dashed line, the start of visual white noise. The left and right gray dashed lines present the start and end of wheel rotation, respectively. **(c)** Similar to (a), but for contralateral-preferring CPNs in CR trials. Black and gray dashed lines, the start and end of visual white noise, respectively. **(d)** Averaged ΔF/F (z-scored) of contralateral-preferring CPNs in CR trials. Colored shading, ±SEM. Black dashed line, the start of visual white noise. Gray shading, window during the presentation of visual white noise. **(e-h)** Similar to (a-b), but for ipsilateral-preferring CPNs. Each row shows the average activity of one neuron, sorted by peak response during the visuomotor period in hit trials (same as showing in Fig. 2i).

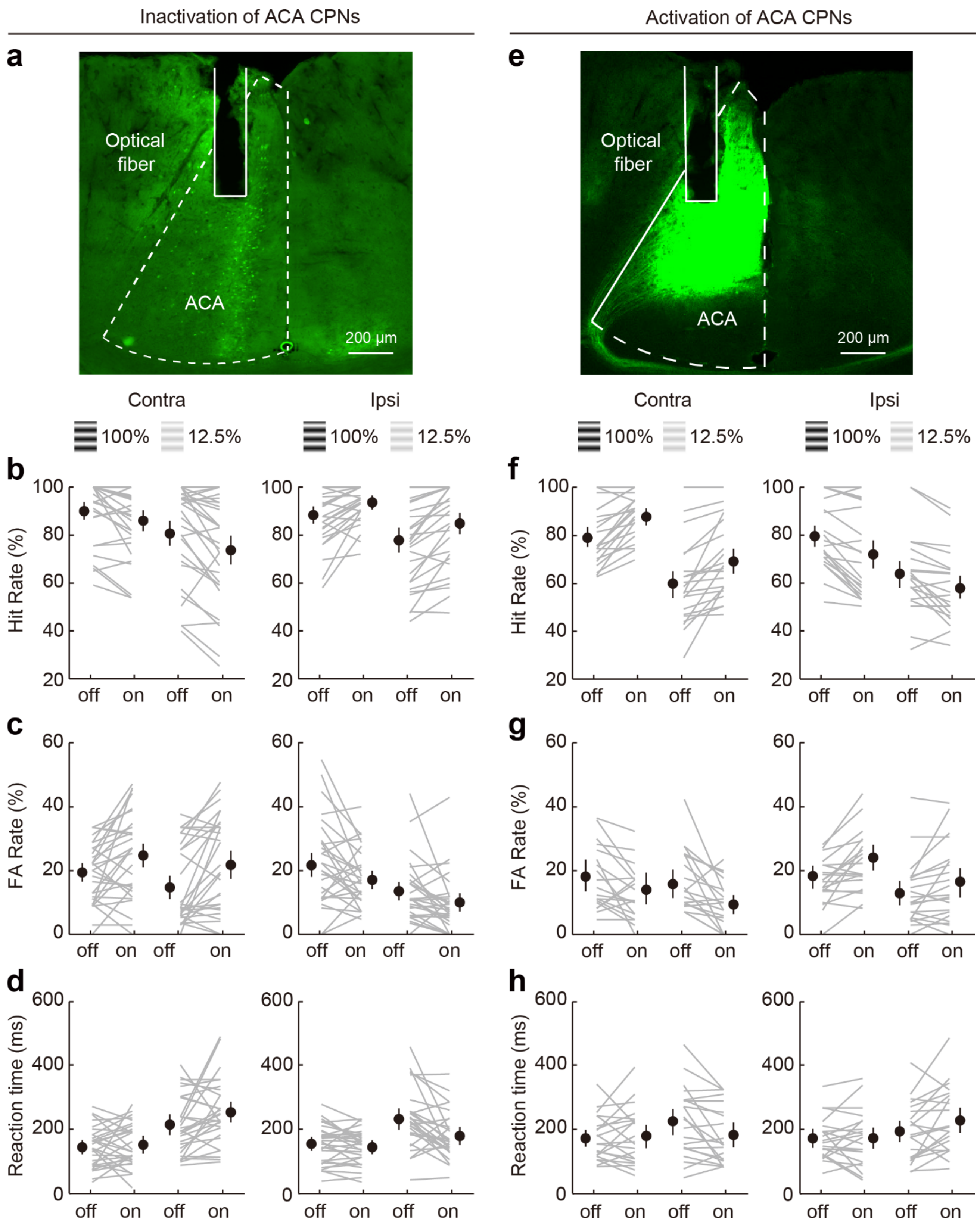

Supplementary Fig. 6. Activation of ACA CPNs causes opposite behavioral changes to inactivation of ACA CPNs in the 2AUC visual change-detection task.

(a) Coronal section showing NpHR fluorescence (green) and the location of implanted optical fiber for unilateral inactivation of ACA CPNs. **(b-d)** Effects of unilateral inactivation of ACA CPNs on hit rates **(b),** FA rates **(c)** and reaction times in hit trials **(e)**. The data shown in **(b-d)** are the same datasets as in Fig. 3c,d, but show laser effects in each session (*n* = 29 sessions). **(e)** Similar to **(a)**, but for showing ChR2 fluorescence (green) and the location of implanted optical fiber for unilateral activation of ACA CPNs. **(f-h)** Similar to **(b-d)**, but for the effects of unilateral activation of ACA CPNs (*n* = 21 sessions). The data shown in **(f-h)** are the same datasets as in Fig. 3e,f. All data are presented as the mean ± SEM.

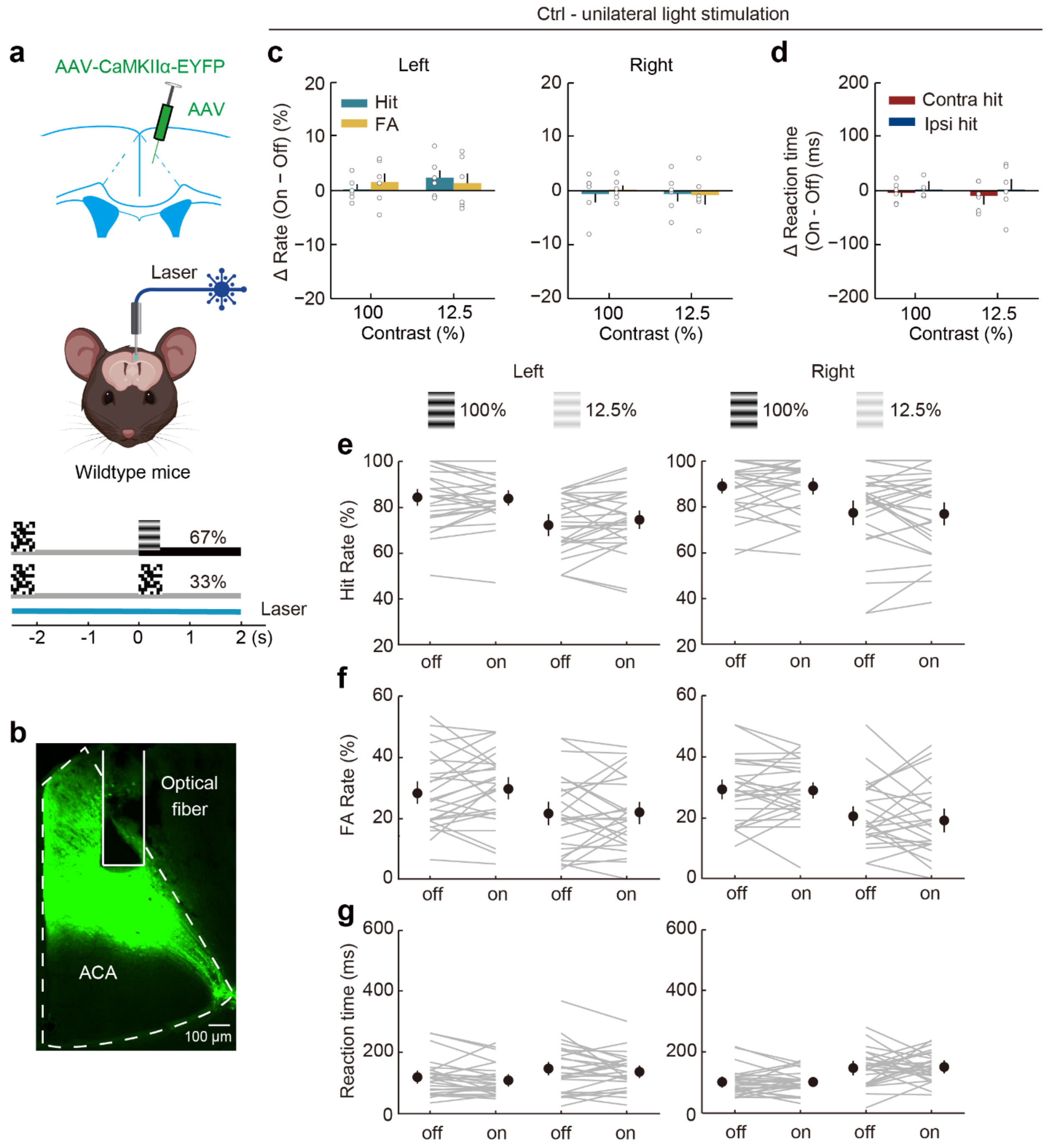

Supplementary Fig. 7. Effects of laser stimulation on the performance of control mice in the 2AUC change-detection task.

(a) Schematic diagrams of the virus injection and unilateral laser stimulation (473 nm, 8 mW, 10 Hz, 5 ms per pulse) in control mice. **(b)** Coronal section showing the locations of virus expression (green) and the implanted optical fiber. **(c-d)** Effect of unilateral laser stimulation in control mice (*n* = 6 mice) on visual change detection (c) and reaction time (d). Blue laser did not induce a change in visual detection or reaction time on either side (hit rates, $F_{side*laser}(1,5) = 1.40$, $P_{side*laser} = 0.29$, $F_{contrast*laser}(1,5) = 0.56$, $P_{contrast*laser} = 0.49$; FA rates, $F_{side*laser}(1,5) = 1.08$, $P_{side*laser} = 0.35$, $F_{contrast*laser}(1,5) = 0.20$, $P_{contrast*laser} = 0.68$; reaction time, $F_{side*laser}(1,5) = 0.67$, $P_{side*laser} = 0.45$, $F_{contrast*laser}(1,5) = 0.04$, $P_{contrast*laser} = 0.85$; three-way repeated ANOVA). **(e-g)** Effects of unilateral laser stimulation on hit

rates **(e)**, FA rates **(f)**, and reaction times in hit trials **(g)**. The data shown in **(e-g)** are the same datasets as in **(c-d)**, but show laser effects in each session (*n* = 27 sessions). All data are presented as the mean ± SEM. Source data are provided as a Source Data file.

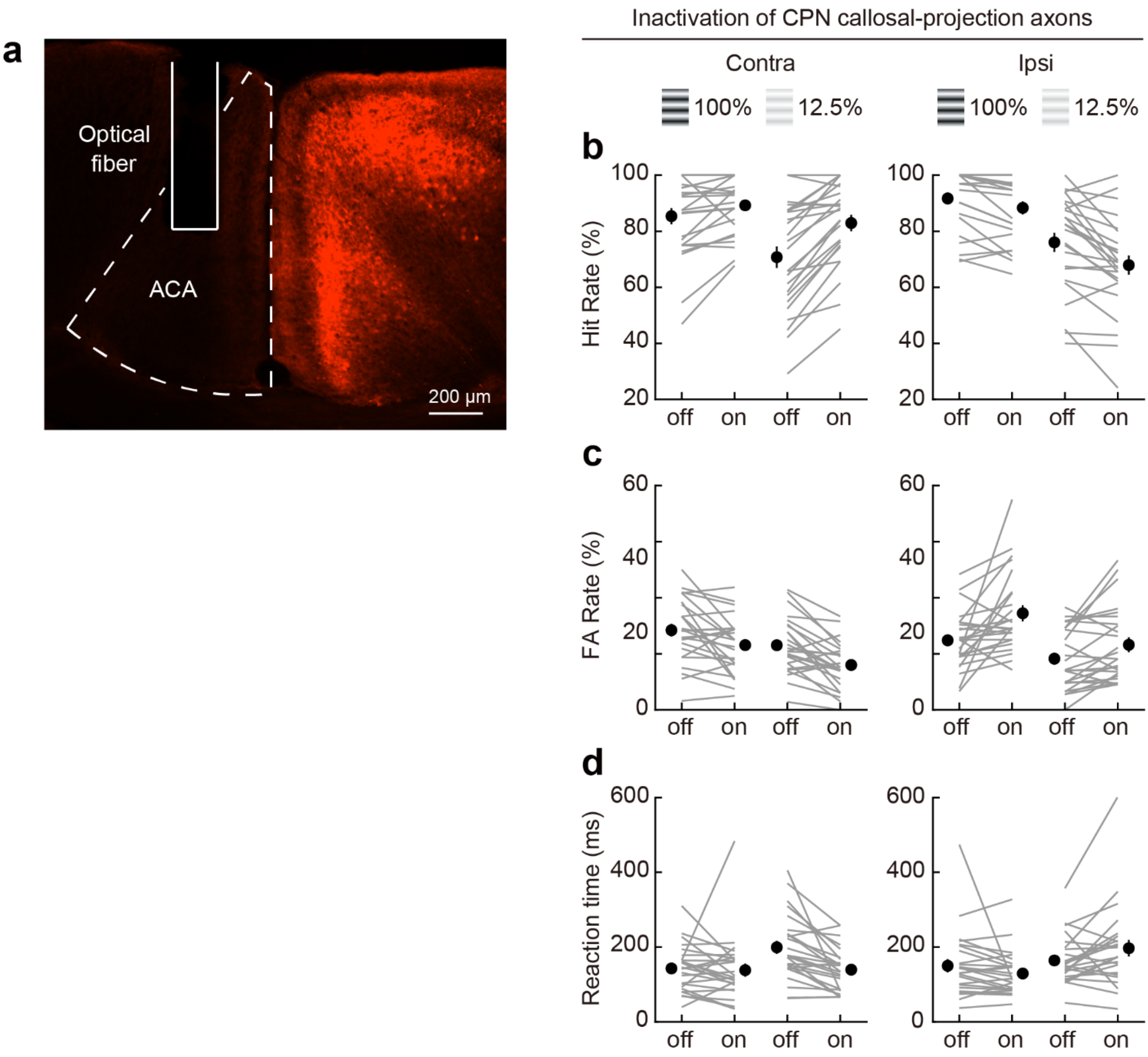

Supplementary Fig. 8. Inactivation of CPN callosal-projection axons causes similar behavioral changes to inactivating their somas.

(a) Coronal section showing eOPN3 fluorescence (red) and the location of implanted optical fiber for inactivation of CPN callosal-projection axons. **(b-d)** Effects of inactivation of CPN callosal-projection axons on hit rates **(b),** FA rates **(c)** and reaction times **(d)** in hit trials. The data shown in **(b-d)** are the same datasets as in Fig. 4e,f, but show laser effects in each session (n = 25 sessions). All data are presented as the mean ± SEM.

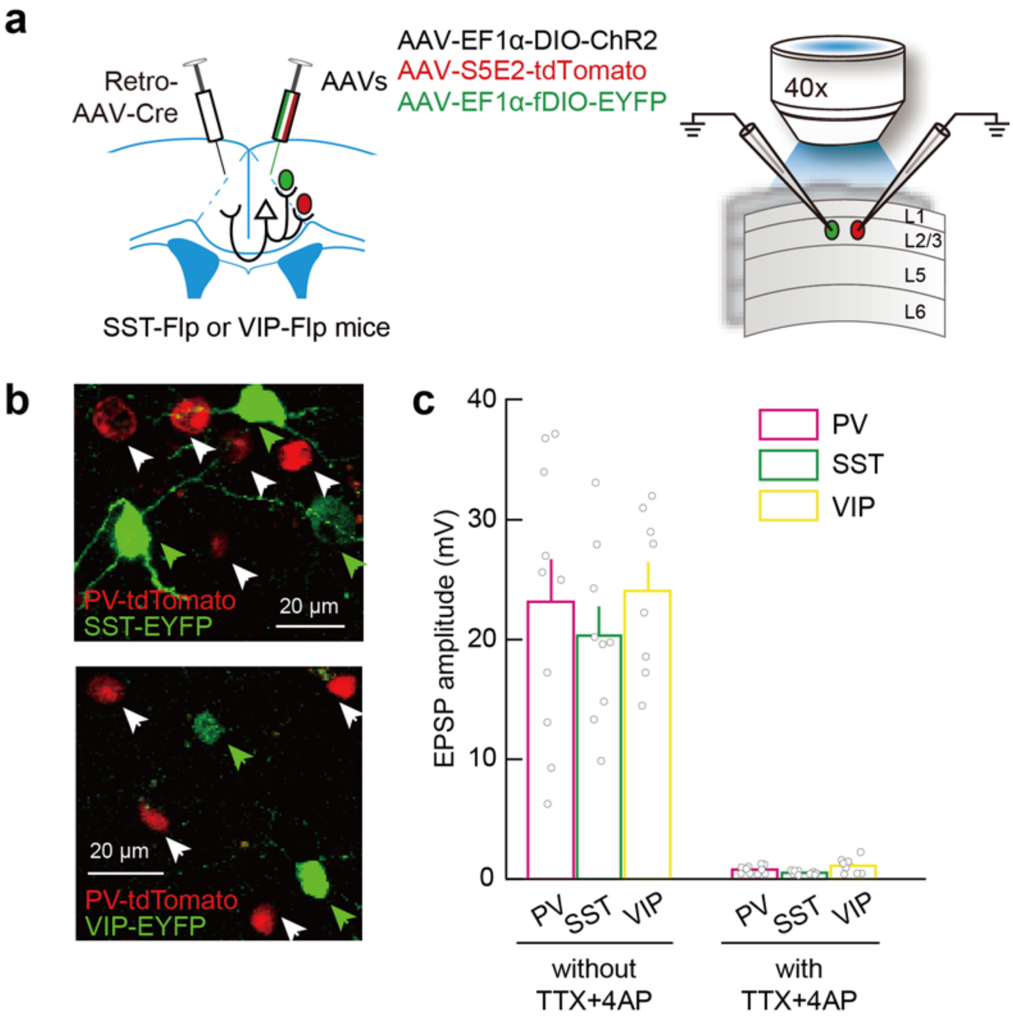

Supplementary Fig. 9. Ipsilateral CPNs provide non-selective and weak direct inputs to three major types of cortical interneurons in the ACA.

(a) Schematic of the slice experiment to measure ipsilateral CPN-induced EPSPs in ACA interneurons. Left, viral vectors and injection procedure for expressing ChR2 in ipsilateral CPNs and fluorescent proteins in cortical interneurons (PV+ neurons, tdTomato; SST+ or VIP+ neurons, EYFP). Right, electrophysiological recording. **(b)** Fluorescence images showing PV+ neurons with enhancer-driven expression of tdTomato (red) and SST+ or VIP+ neurons with fDIO-induced expression of EYFP (green). Top, red arrowheads, tdTomato-expressing PV+ neurons; white arrowheads, EYFP-expressing SST+ neurons. Bottom, red arrowheads, tdTomato-expressing PV+ neurons; white arrowheads, EYFP-expressing VIP+ neurons. **(c)** Ipsilateral CPN-induced polysynaptic (without TTX+4-AP) and monosynaptic (with TTX+4-AP) EPSP amplitudes in different cell types. The ipsilateral CPN-induced monosynaptic EPSPs are weak and non-selective across three major types of cortical interneurons (PV+ *vs.* SST+, P = 0.08; PV+ *vs.* VIP+, P = 0.21; SST+ *vs.* VIP+, P = 0.07; Wilcoxon rank-sum test). All data are presented as the mean ± SEM. Source data are provided as a Source Data file.

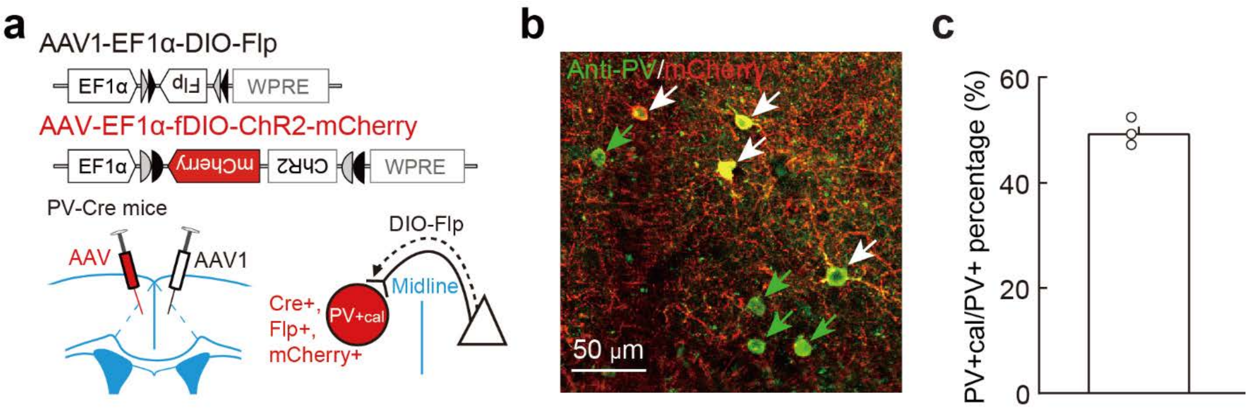

Supplementary Fig. 10. Percentage of PV+$_{cal}$ neurons in all PV+ neurons.

(a) Viral vectors and injection procedure for identification of callosal-input-driven PV+$_{cal}$ neurons. Anterograde Cre-inducible AAV1 expressing Flp (AAV1-EF1α-DIO-Flp) was injected into the ACA of one hemisphere, and Flp-dependent AAV expressing mCherry (AAV-EF1α-fDIO-ChR2-mCherry) was injected into the opposite ACA of PV-Cre mice. **(b)** Example fluorescence image showing mCherry-expressing PV+$_{cal}$ neurons (red) and PV immunostaining (green) in the ACA. White arrowheads, PV+$_{cal}$ neurons, which are PV+ and mCherry+. Green arrowheads, PV+ neurons without mCherry expression. **(c)** Quantification of the percentage of PV+$_{cal}$ neurons (PV+ and mCherry+) in all PV+ neurons (n = 3 mice). All data are presented as mean ± SEM. Source data are provided as a Source Data file.

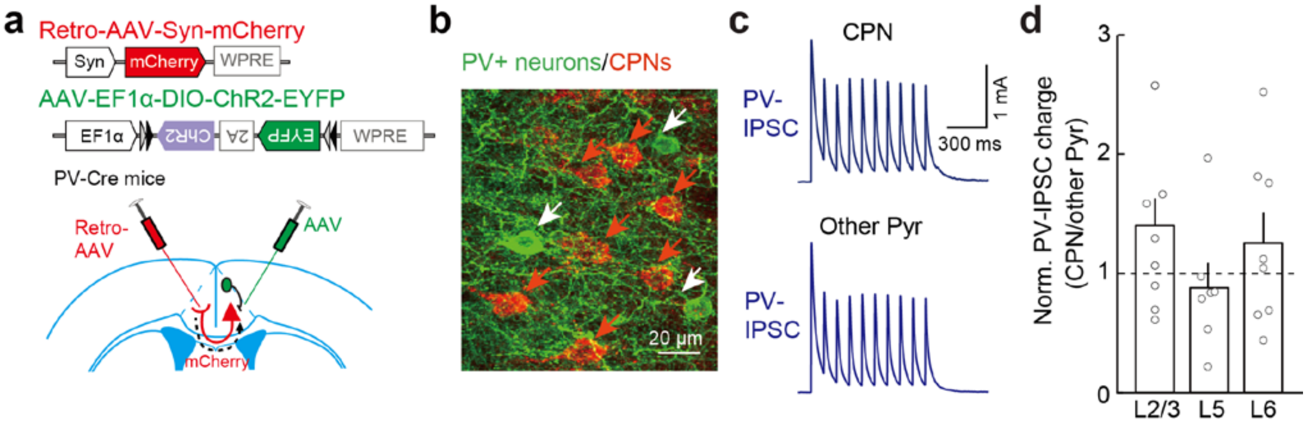

Supplementary Fig. 11. PV+ neurons induced similar inhibition of CPNs and other Pyramidal neurons.

(a) Viral vectors and injection procedure for identification of ACA CPNs and optogenetic activation of PV+ neurons. **(b)** Example fluorescence image showing ChR2-EYFP-expressing PV+ neurons (green) and mCherry-expressing CPNs (red) in the ACA. White arrowheads, PV+ neurons. Red arrowheads, CPNs. **(c)** Example PV-IPSCs recorded from a layer 5 CPN (top) and a nearby Pyr (bottom). **(d)** Normalized PV-IPSC charge. Activation of all PV+ neurons induced similar IPSCs in CPNs and other pyramidal neurons (P > 0.1, Wilcoxon signed-rank test). All data are presented as mean ± SEM. Source data are provided as a Source Data file.

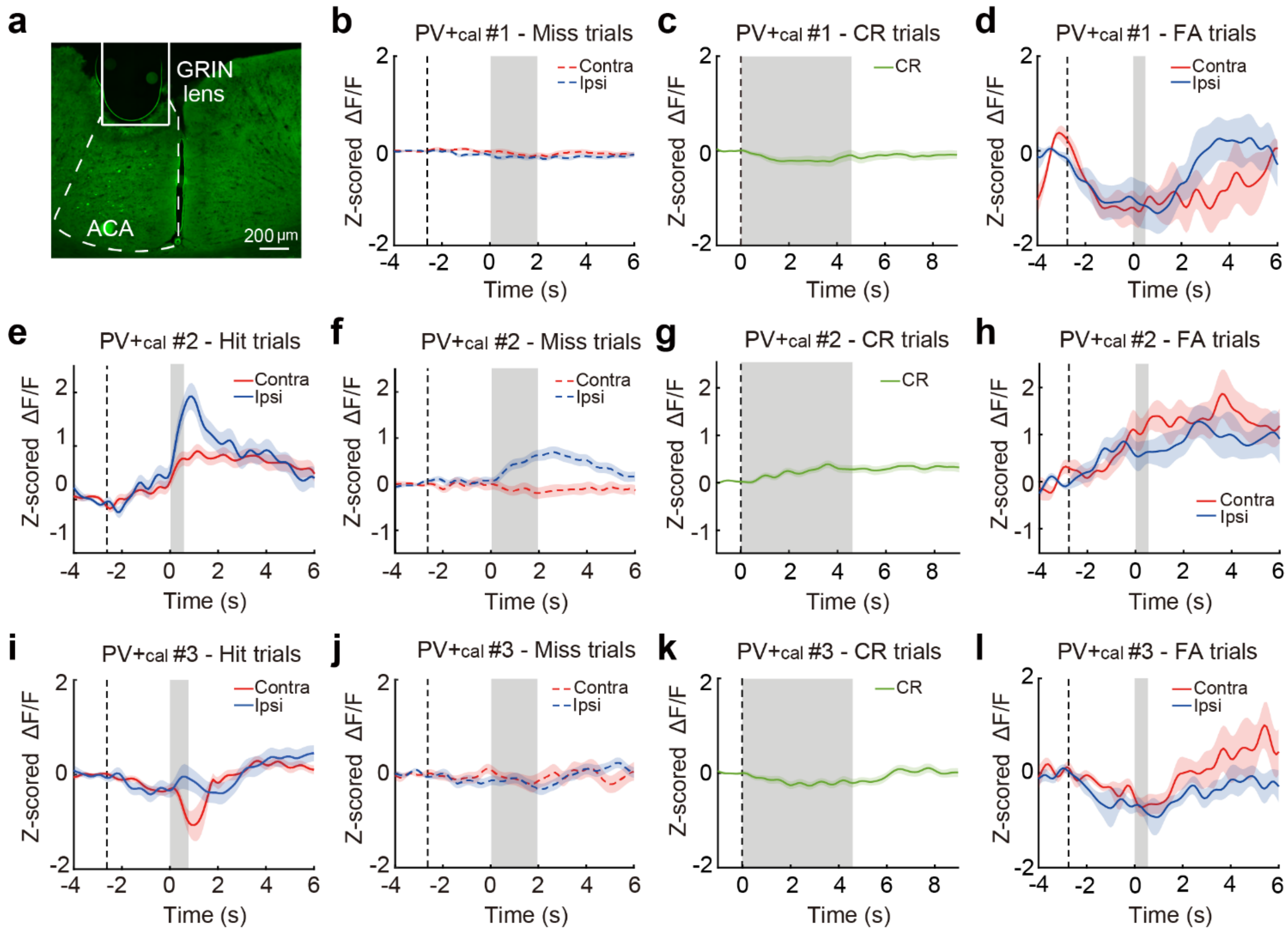

Supplementary Fig. 12. The activity of example PV+$_{cal}$ neurons in the 2AUC change-detection task.

(a) Coronal section showing GCaMP6s fluorescence (green) and the location of implanted GRIN lens. **(b-d)** Averaged ΔF/F (z-scored) of PV+$_{cal}$ #1. Dashed line, the start of visual white noise. Colored shading, ±SEM. **(b)** Activity in miss trials. Gray shading, window during the presentation of drifting grating. **(c)** Activity in CR trials. Gray shading, window during the presentation of visual white noise. **(d)** Activity in FA trials. Gray shading, window during the wheel rotation. **(e-h)** Averaged ΔF/F (z-scored) of PV+$_{cal}$ #2. **(e)** Activity in hit trials. Gray shading, window from the start of drifting grating to the end of wheel rotation. **(f-h)** Similar to **(b-d)**, but for PV+$_{cal}$ #2. **(i-l)** Similar to **(e-h)**, but for PV+$_{cal}$ #3.

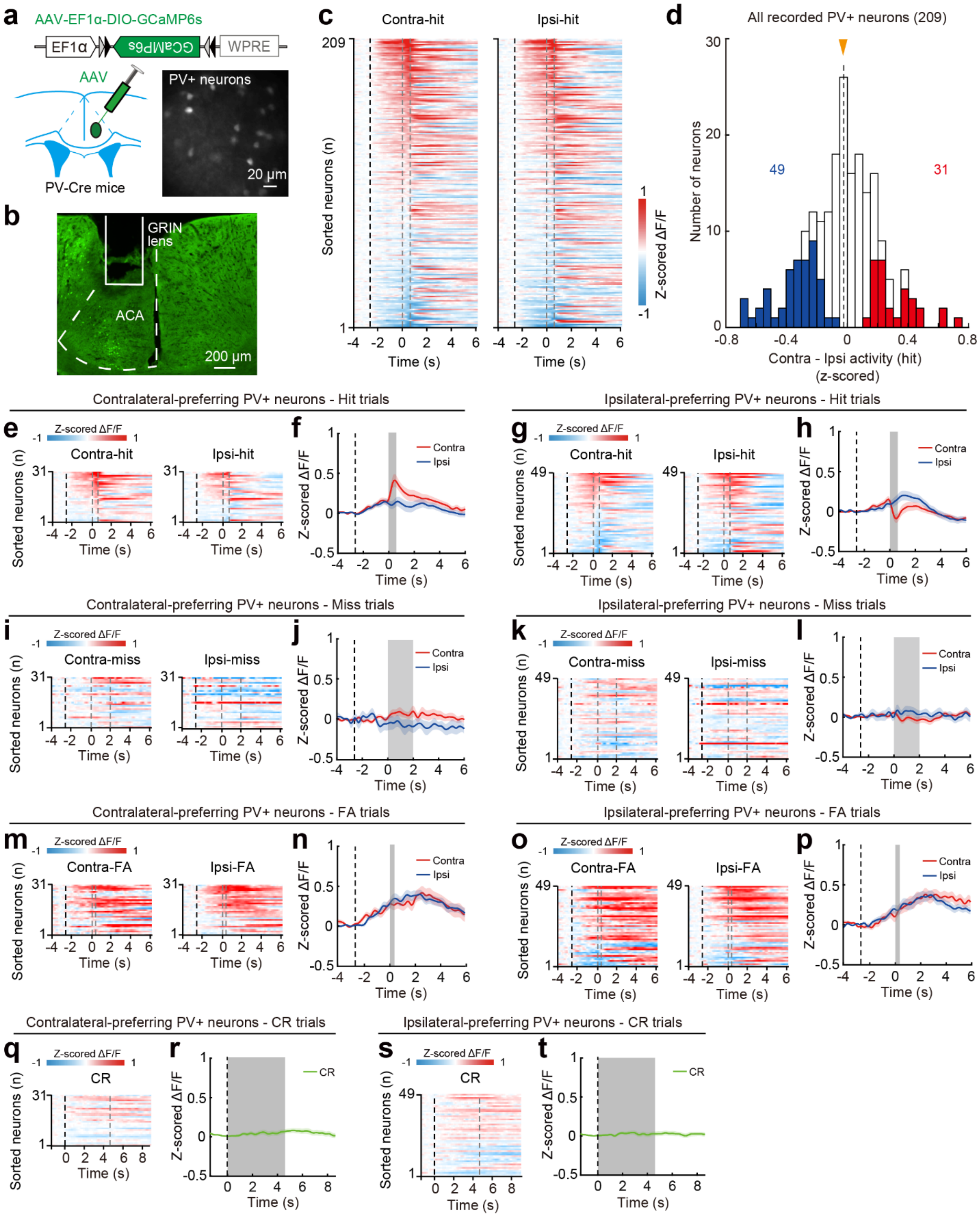

Supplementary Fig. 13. The activity of PV+ neurons in the 2AUC change-detection task.
(a) Schematic diagrams for viral strategy (left) and calcium imaging of PV+ neurons (right). **(b)** Coronal section showing GCaMP6s fluorescence (green) and the location of implanted GRIN lens. **(c)** Color-coded averaged ΔF/F (z-scored) of all recorded PV+ neurons in contralateral-hit (left) and ipsilateral-hit (right) trials (n = 7 mice, 7 sessions, 209 neurons). Each row shows the average activity

of one neuron, sorted by peak response during the visuomotor period. Black dashed line, the start of visual white noise. The left and right gray dashed lines present the start of drifting grating and the end of wheel rotation, respectively. **(d)** Distribution of the difference in peak responses of z-scored ΔF/F during the visuomotor period between the contralateral-hit and ipsilateral-hit trials in the imaged PV+ neurons. Blue, ipsilateral-preferring cells; red, contralateral-preferring cells. Orange arrowhead, median. **(e)** Similar to **(c)**, but for contralateral-preferring PV+ neurons. **(f)** Averaged ΔF/F (z-scored) of contralateral-preferring PV+ neurons in hit trials. Colored shading, ±SEM. Dashed line, the start of visual white noise. Gray shading, window of the visuomotor period. **(g-h)** Similar to **(e-f)** but for ipsilateral-preferring PV+ neurons in hit trials. **(i-j)** Similar to **(e-f)**, but for contralateral-preferring PV+ neurons in miss trials. **(i)** Black dashed line, the start of visual white noise. The left and right gray dashed lines present the start and end of drifting grating, respectively. **(j)** Black dashed line, the start of visual white noise. Gray shading, window during the presentation of drifting grating. **(k-l)** Similar to **(i-j)** but for ipsilateral-preferring PV+ neurons in miss trials. **(m-n)** Similar to **(e-f)**, but for contralateral-preferring PV+ neurons in FA trials. **(m)** Black dashed line, the start of visual white noise. The left and right gray dashed lines present the start and end of wheel rotation, respectively. **(n)** Black dashed line, the start of visual white noise. Gray shading, window during the wheel rotation. **(o-p)** Similar to **(m-n)**, but for ipsilateral-preferring PV+ neurons in FA trials. **(q-r)** Similar to **(e-f)**, but for contralateral-preferring PV+ neurons in CR trials. **(q)** Black and gray dashed lines, the start and end of visual white noise, respectively. **(r)** Black dashed line, the start of visual white noise. Gray shading, window during the presentation of visual white noise. **(s-t)** Similar to **(q-r)**, but for ipsilateral-preferring PV+ neurons in CR trials. Source data are provided as a Source Data file.

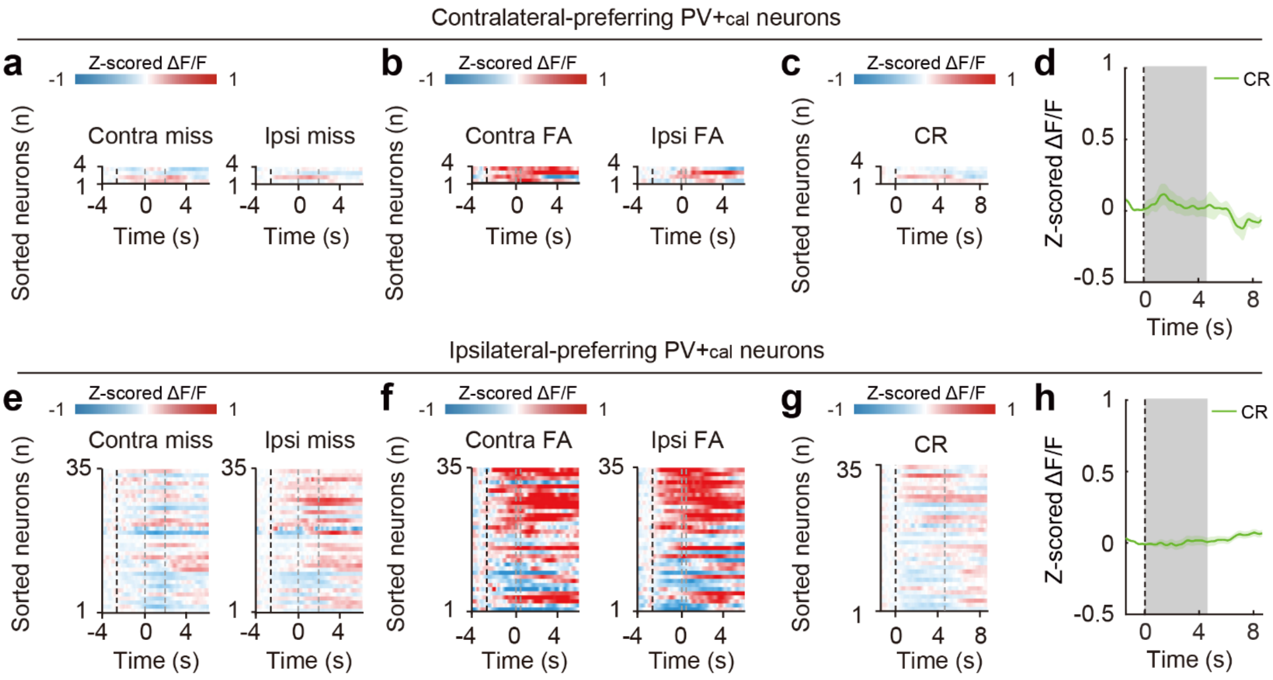

Supplementary Fig. 14. The activity of contralateral-preferring and ipsilateral-preferring PV+$_{cal}$ neurons in the 2AUC change-detection task.

(a) Color-coded averaged ΔF/F (z-scored) of contralateral-preferring PV+$_{cal}$ neurons in miss trials. Each row shows the average activity of one neuron, sorted by peak response during the visuomotor period in hit trials (same as showing in Fig. 7g). Black dashed line, the start of visual white noise. The left and right gray dashed lines present the start and end of drifting grating, respectively. **(b)** Similar to (a), but for contralateral-preferring PV+$_{cal}$ neurons in FA trials. Black dashed line, the start of visual white noise. The left and right gray dashed lines present the start and end of wheel rotation, respectively. **(c)** Similar to (a), but for contralateral-preferring PV+$_{cal}$ neurons in CR trials. Black and gray dashed lines, the start and end of visual white noise, respectively. **(d)** Averaged ΔF/F (z-scored) of contralateral-preferring PV+$_{cal}$ neurons in CR trials. Colored shading, ±SEM. Black dashed line, the start of visual white noise. Gray shading, window during the presentation of visual white noise. **(e-h)** Similar to (a-b), but for ipsilateral-preferring PV+$_{cal}$ neurons. Each row shows the average activity of one neuron, sorted by peak response during the visuomotor period in hit trials (same as showing in Fig. 7i).

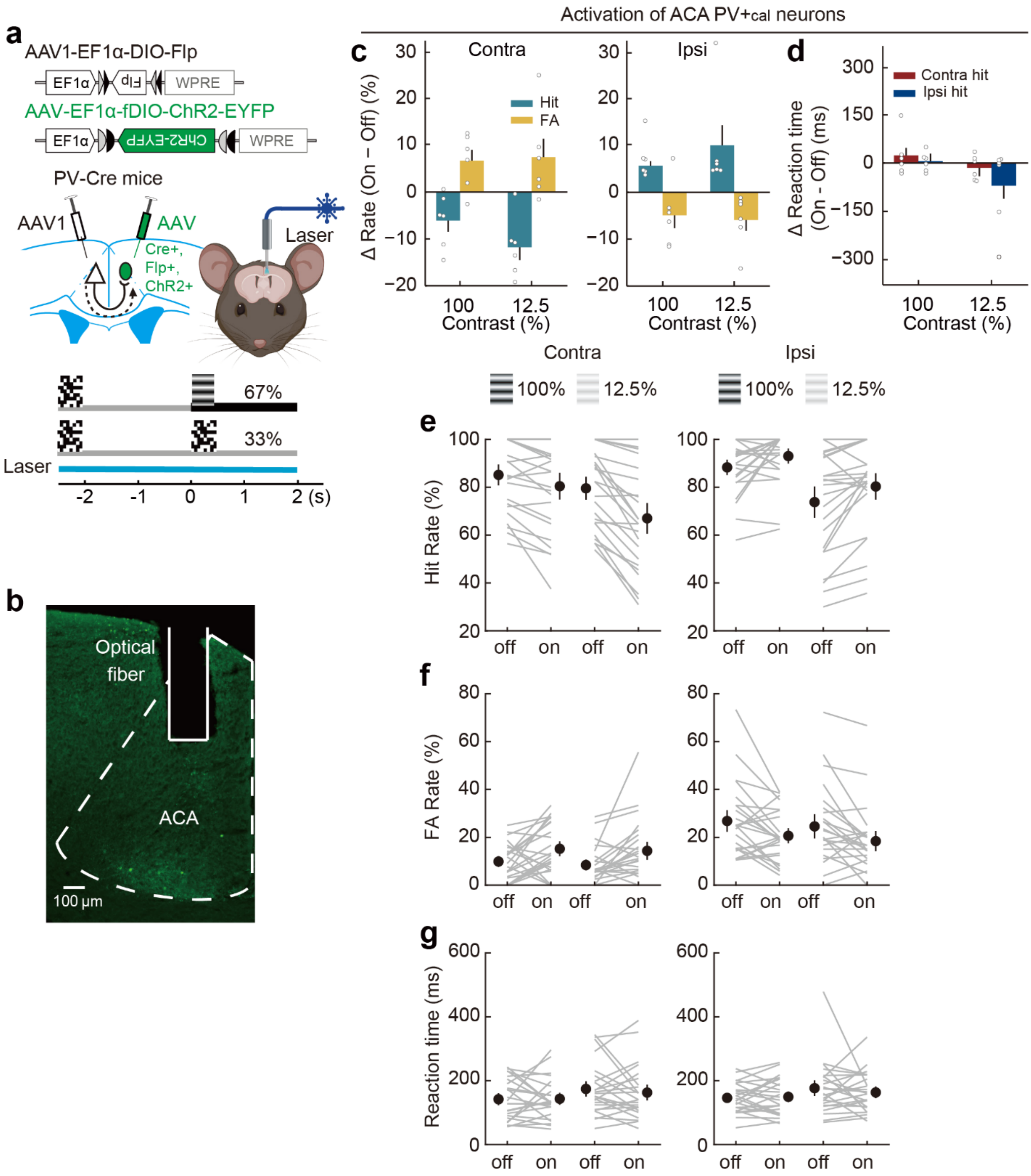

Supplementary Fig. 15. Effects of unilateral activation of PV+$_{cal}$ neurons on the performance in the 2AUC change-detection task.

(a) Schematic diagrams of the viral strategy (top) and optogenetic activation of PV+$_{cal}$ neurons (bottom). **(b)** Coronal section showing the location of implanted optical fiber. **(c)** Effect of unilateral activation of PV+$_{cal}$ neurons on visual change detection (*n* = 6 mice). Left, changes in contralateral performance. Right, changes in ipsilateral performance. Unilateral activation of PV+$_{cal}$ neurons (blue laser, 473 nm, 1-5 mW, 10 Hz, 5 ms per pulse) had similar effects on hit rates and FA rates at the 100% and 12.5% contrast levels (hit rates, $F_{contrast*laser}(1,5)$ = 0.12, $P_{contrast*laser}$ = 0.75; FA rates, $F_{contrast*laser}(1,5)$ = 0.004, $P_{contrast*laser}$ = 0.95; three-way repeated ANOVA), while having significantly different effects on contralateral vs. ipsilateral hit rates and FA rates (hit rates, $F_{side*laser}(1,5)$ = 54.48,

$P_{side*laser}$ = 0.001; FA rates, $F_{side*laser}(1,5)$ = 56.80, $P_{side*laser}$ = 0.001; three-way repeated ANOVA). Unilateral activation of PV+$_{cal}$ neurons significantly impaired contralateral detection (decreased hit rates, green bars, $F_{laser}(1,5)$ = 17.22, P_{laser} = 0.009; increased FA rates, yellow bars, $F_{laser}(1,5)$ = 7.55, P_{laser} = 0.04; two-way repeated ANOVA) and improved ipsilateral detection (increased hit rates, green bars, $F_{laser}(1,5)$ = 14.65, P_{laser} = 0.01; decreased FA rates, yellow bars, $F_{laser}(1,5)$ = 8.26, P_{laser} = 0.035; two-way repeated ANOVA). **d**, Effect of unilateral activation of PV+$_{cal}$ neurons on reaction times in hit trials. Unilateral activation of PV+$_{cal}$ neurons did not cause significant change in the reaction time (reaction time, $F_{side*laser}(1,5)$ = 1.15, $P_{side*laser}$ = 0.33, $F_{contrast*laser}(1,5)$ = 2.29, $P_{contrast*laser}$ = 0.19; three-way repeated ANOVA). **(e-g)** Effects of unilateral laser stimulation on hit rates **(e)**, FA rates **(f)** and reaction times in hit trials **(g)**. The data shown in **(e-g)** are the same datasets as in **(c-d)**, but show laser effects in each session (*n* = 24 sessions). All data are presented as the mean ± SEM. Also see Supplementary Data 2 for detailed parameters of ANOVA. Source data are provided as a Source Data file.

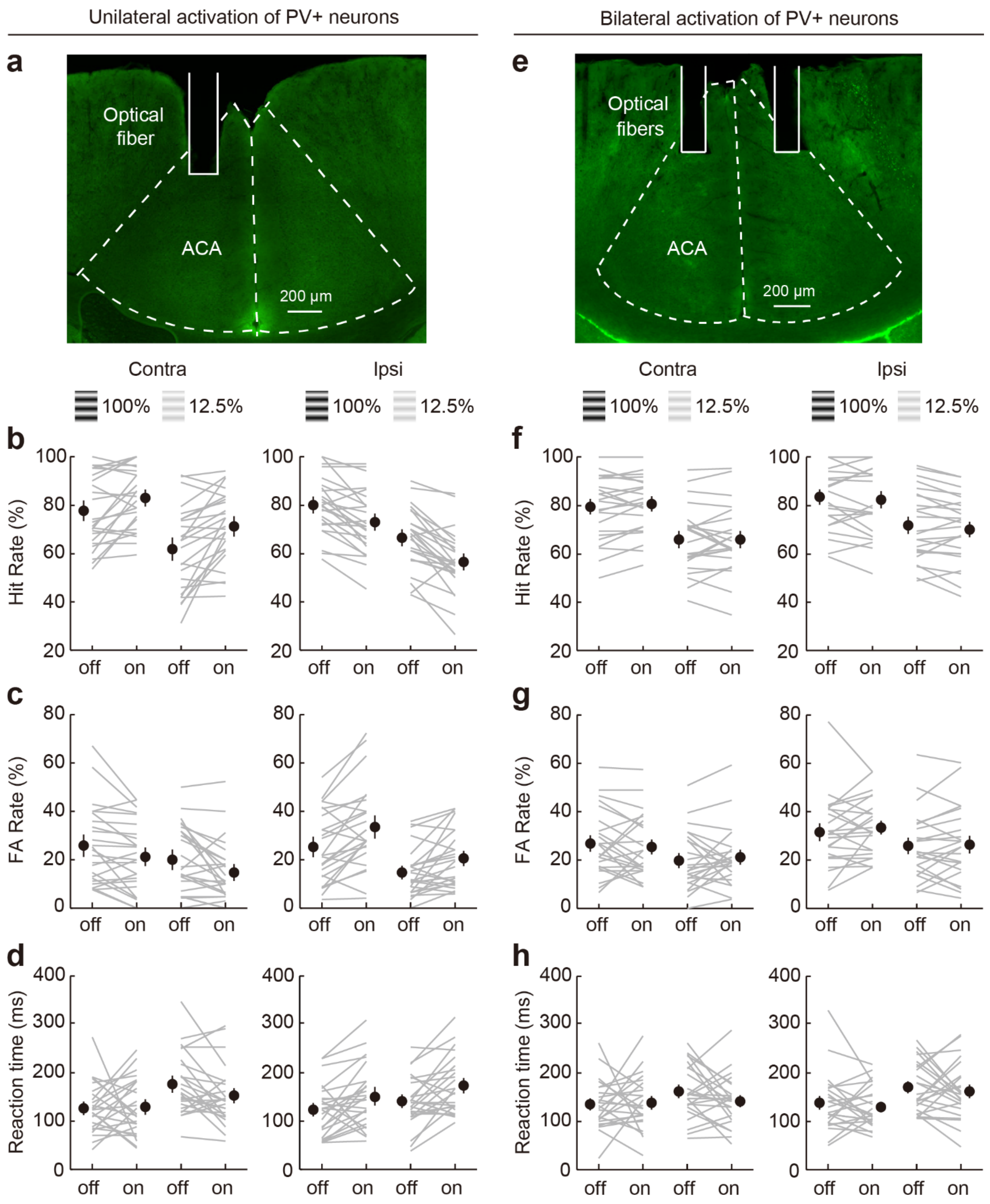

Supplementary Fig. 16. Bilateral activation of PV+ neurons reversed the behavioral bias induced by unilateral activation of PV+ neurons.

(a) Coronal section showing the location of implanted optical fiber for unilateral activation of PV+ neurons in PV-Ai32 mice. **(b-d)** Effects of unilateral activation of PV+ neurons on hit rates **(b),** FA

rates **(c)** and reaction times in hit trials **(e)**. The data shown in **(b-d)** are the same datasets as in Fig. 8b,c, but show laser effects in each session (*n* = 25 sessions). **(e)** Similar to **(a)**, but for showing the location of implanted optical fibers for bilateral activation of PV+ neurons in PV-Ai32 mice. **(f-h)** Similar to **(b-d)**, but for the effects of bilateral activation of PV+ neurons (*n* = 24 sessions). The data shown in **(f-h)** are the same datasets as in Fig. 8d,e. All data are presented as the mean ± SEM.

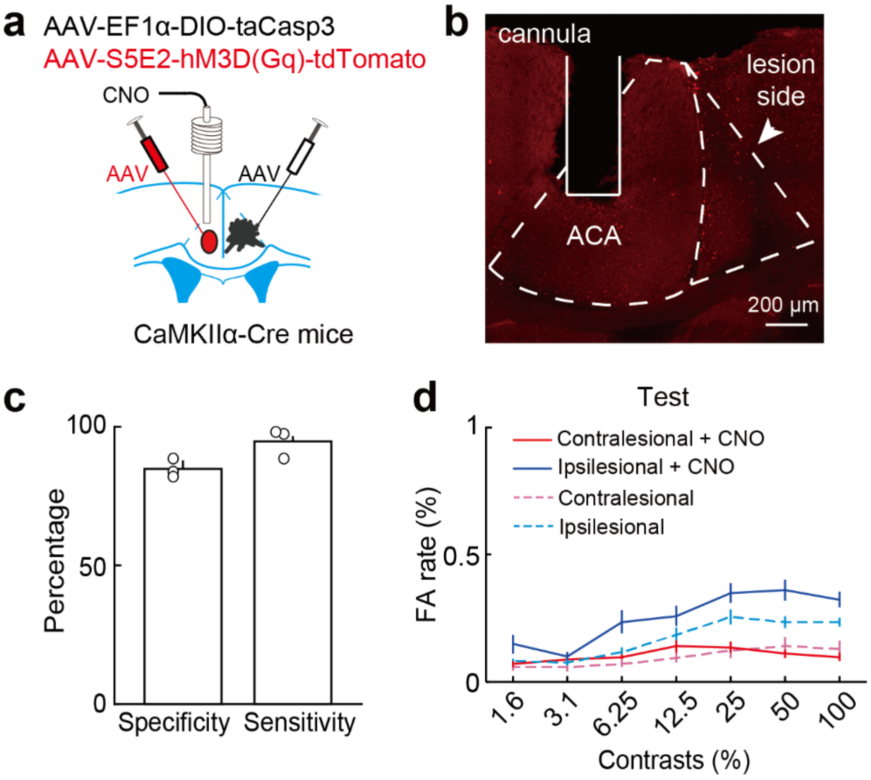

Supplementary Fig. 17. Enhancer-driven hM3D(Gq) expression in PV+ neurons in CaMKIIα-Cre mice.

(a) Schematic of the viral strategy for enhancer-driven expression of hM3D(Gq) in PV+ neurons in the contralesional ACA. **(b)** Coronal section showing an ACA lesion (right), contralesional hM3D fluorescence (red), and the location of the implanted cannula (left). We noted that the ipsilesional ACA and the area near the implanted cannula showed a relatively strong spontaneous fluorescence signal, which could represent dead cell debris. **(c)** Specificity and sensitivity of enhancer-driven expression of hM3D(Gq)-tdTomato in PV+ neurons. "Specificity" here refers to the percentage of cells expressing the tdTomato (red) that co-express PV (anti-PV, green, see Fig. 8f); "Sensitivity" refers to the percentage of cells expressing PV (anti-PV, green) that co-express tdTomato (red). **(d)** FA rates of ACA-lesioned mice in test phase with or without contralesional PV+ neuron activation during learning phase1. In CNO-treated ACA-lesioned mice, ipsilesional FA rates were significantly higher than contralesional FA rates (n = 6 mice, $F_{side}(1,5) = 31.32$, $P_{side} = 0.003$; two-way repeated ANOVA). The ipsilesional FA rates in CNO-treated ACA-lesioned mice were significantly lower than in the control ACA-lesioned group (*i.e.*, without treatment) ($F_{CNO}(1,105) = 113.45$, $P_{CNO} = 2 \times 10^{-18}$, two-way ANOVA). The contralesional FA rates in CNO-treated ACA-lesioned mice were similar to those detected for the control ACA-lesioned group ($F_{CNO}(1,105) = 0.41$, $P_{CNO} = 0.52$, two-way ANOVA). The data shown with dashed lines are the same datasets as in Fig. 1j. All data are presented as the mean ± SEM. Also see Supplementary Data 2 for detailed parameters of ANOVA. Source data are provided as a Source Data file.
