# Supplementary material for: A frontal transcallosal inhibition loop mediates interhemispheric balance in visuospatial processing
Source: Nat Commun. 2023 Aug 25;14:5213. doi: 10.1038/s41467-023-40985-5 (PMC10457336; doi:10.1038/s41467-023-40985-5)
Supplement: Supplementary file 3 — Description of Additional Supplementary Files [file 41467_2023_40985_MOESM3_ESM.pdf]

**Description of Additional Supplementary Files**

File Name: Supplementary Data 1
Description: Effects of unilateral inactivation and activation of ACA CPNs, CPN callosal-projection axons, PV+ neurons and $PV+_{cal}$ neurons on behavioral performance in 2AUC change-detection task.

File Name: Supplementary Data 2
Description: The details of ANOVA.
