# Supplementary material for: A frontal transcallosal inhibition loop mediates interhemispheric balance in visuospatial processing
Source: Nat Commun. 2023 Aug 25;14:5213. doi: 10.1038/s41467-023-40985-5 (PMC10457336; doi:10.1038/s41467-023-40985-5)
Supplement: Supplementary file 6 — Reporting Summary [file 41467_2023_40985_MOESM6_ESM.pdf]

nature portfolio

Corresponding author(s): Min Xu, Siyu Zhang

Last updated by author(s): Aug 12, 2023

Reporting Summary

Nature Portfolio wishes to improve the reproducibility of the work that we publish. This form provides structure for consistency and transparency in reporting. For further information on Nature Portfolio policies, see our Editorial Policies and the Editorial Policy Checklist.

Statistics

For all statistical analyses, confirm that the following items are present in the figure legend, table legend, main text, or Methods section.

n/a	Confirmed	
☐	☒	The exact sample size (*n*) for each experimental group/condition, given as a discrete number and unit of measurement
☐	☒	A statement on whether measurements were taken from distinct samples or whether the same sample was measured repeatedly
☐	☒	The statistical test(s) used AND whether they are one- or two-sided<br>*Only common tests should be described solely by name; describe more complex techniques in the Methods section.*
☒	☐	A description of all covariates tested
☐	☒	A description of any assumptions or corrections, such as tests of normality and adjustment for multiple comparisons
☐	☒	A full description of the statistical parameters including central tendency (e.g. means) or other basic estimates (e.g. regression coefficient) AND variation (e.g. standard deviation) or associated estimates of uncertainty (e.g. confidence intervals)
☐	☒	For null hypothesis testing, the test statistic (e.g. *F*, *t*, *r*) with confidence intervals, effect sizes, degrees of freedom and *P* value noted<br>*Give P values as exact values whenever suitable.*
☒	☐	For Bayesian analysis, information on the choice of priors and Markov chain Monte Carlo settings
☒	☐	For hierarchical and complex designs, identification of the appropriate level for tests and full reporting of outcomes
☒	☐	Estimates of effect sizes (e.g. Cohen's *d*, Pearson's *r*), indicating how they were calculated

Our web collection on statistics for biologists contains articles on many of the points above.

Software and code

Policy information about availability of computer code

Data collection	The codes used for behavioral data and calcium imaging collection were developed with Matlab (2014b).<br>The softwares and codes used for anatomic data collection in this study were similar to previous studies (Zhang et al. 2016. Nat. Neurosci.; Ma et al. 2021. Sci. Adv.) and described in Supplementary Materials and Methods.<br>The softwares and codes used for slice physiology were described elsewhere (Zhang et al. 2014. Science; Ma et al. 2021. Sci. Adv.). Axon pCLAMP 10 was used for physiological data collection.
Data analysis	Data were analyzed and visualized with MatLab (2014b). ANOVA were conducted with SPSS (IBM SPSS Statistics 25). Adobe Illustrator CS6 were used in the production of data figures.

For manuscripts utilizing custom algorithms or software that are central to the research but not yet described in published literature, software must be made available to editors and reviewers. We strongly encourage code deposition in a community repository (e.g. GitHub). See the Nature Portfolio guidelines for submitting code & software for further information.

Data

Policy information about availability of data

All manuscripts must include a data availability statement. This statement should provide the following information, where applicable:

- Accession codes, unique identifiers, or web links for publicly available datasets
- A description of any restrictions on data availability
- For clinical datasets or third party data, please ensure that the statement adheres to our policy

All data needed to evaluate the conclusions in the paper are present in the paper and the Supplementary Materials. Data related to this paper may be requested from the authors. Illustrations containing drawings of mice were created with BioRender.com. The Allen 3D mouse brain atlas, which is used for anatomical data analysis, can be accessed at mouse.brain-map.org and atlas.brain-map.org. Source data are provided with this paper.

Human research participants

Policy information about studies involving human research participants and Sex and Gender in Research.

Reporting on sex and gender	N/A
Population characteristics	N/A
Recruitment	N/A
Ethics oversight	N/A

Note that full information on the approval of the study protocol must also be provided in the manuscript.

Field-specific reporting

Please select the one below that is the best fit for your research. If you are not sure, read the appropriate sections before making your selection.

☒ Life sciences ☐ Behavioural & social sciences ☐ Ecological, evolutionary & environmental sciences

For a reference copy of the document with all sections, see nature.com/documents/nr-reporting-summary-flat.pdf

Life sciences study design

All studies must disclose on these points even when the disclosure is negative.

Sample size	No statistical methods were used to predetermine sample size, but the sample size was similar to previous studies (Ährlund-Richter et al., 2019, Nat. Neurosci.; Sun et al., 2019, Nat. Neurosci.; Kim et al., 2021, Nat. Neurosci.; Lee et al., 2012, Nature).
Data exclusions	For anatomic data analysis, experimental animals with suboptimal targeting of starter neurons were excluded.<br>For physiological data analysis, data exclusions were based on the quality of recordings. The recordings were excluded when they terminated too shortly or the series resistance became too high.<br>For behavioral data analysis, mice that couldn't learn the task were excluded.
Replication	The exact number of repetitions (individual data points from separate cells and/or animal) are indicated in figures and legends. Reproducibility was ensured by sampling from multiple biological replicates, i.e., from multiple mice. No results were included that were not observed in multiple animals.
Randomization	Animals were not randomized due to the necessity of a genetic construct.
Blinding	Investigators were not blind to subject groups because knowledge of experimental conditions was required during data collection and evaluation.

Reporting for specific materials, systems and methods

We require information from authors about some types of materials, experimental systems and methods used in many studies. Here, indicate whether each material, system or method listed is relevant to your study. If you are not sure if a list item applies to your research, read the appropriate section before selecting a response.

Materials & experimental systems

n/a	Involved in the study	
☐	☒	Antibodies
☒	☐	Eukaryotic cell lines
☒	☐	Palaeontology and archaeology
☐	☒	Animals and other organisms
☒	☐	Clinical data
☒	☐	Dual use research of concern

Methods

n/a	Involved in the study	
☒	☐	ChIP-seq
☒	☐	Flow cytometry
☒	☐	MRI-based neuroimaging

Antibodies

Antibodies used

Lesion experiment: NeuroTrace, Thermo Fisher Scientific(cat# N-21480) , 1:200.
Immunohistochemistry for PV and mCherry/tdTomato experiment: Primary antibody: anti-PV guinea pig polyclonal antibody, Synaptic Systems(cat# 195004), 1:500, and anti-DsRed rabbit polyclonal antibody, TAKARA(cat# 632496), 1:500. Second antibody: Alexa Fluor 647 goat anti-guinea pig IgG, Thermo Fisher Scientific(cat# A-21450), 1:1000, and alexa Fluor 594 donkey anti-rabbit IgG, Thermo Fisher Scientific(cat# A-21207), 1:750.

Validation

Antibodies were validated by the manufacturer-information obtained from the manufacturer's website. For Neurotrace (https://assets.thermofisher.cn/TFS-Assets/LSG/manuals/mp21480.pdf), anti-PV (https://www.sysy.com/product-factsheet/SySy_195004) anti-mCherry and tdtomato (https://www.takarabio.com/documents/Certificate%20of%20Analysis/632496/632496-101717.pdf)

Animals and other research organisms

Policy information about studies involving animals; ARRIVE guidelines recommended for reporting animal research, and Sex and Gender in Research

Laboratory animals

We used both wildtype mice (C57) and transgenic mice. The transgenic mouse lines included PV-Cre (Jackson lab stock #017320), SST-Cre (#013044), VIP-Cre aged (#010908), CaMKIIα-Cre (#005359), loxP-flanked-tdTomato (#007909), SST-Flp (#031629), VIP-Flp (#028578) and loxP-flanked-ChR2-EYFP (#024109) mice. The mice used were aged between 1.5 and 6 months.

Wild animals

This study did not involve wild animals.

Reporting on sex

Male and female mice were used.

Field-collected samples

This study did not involve samples collected from the field.

Ethics oversight

Animal care and the experimental protocols were approved by the Animal Committee of Shanghai Jiao Tong University School of Medicine and the Animal Committee of the Institute of Neuroscience, Chinese Academy of Sciences.

Note that full information on the approval of the study protocol must also be provided in the manuscript.
